# Assessing factors influencing return back to work after cholecystectomy: a qualitative research

**DOI:** 10.1186/1471-230X-10-12

**Published:** 2010-01-27

**Authors:** Frederik Keus, Jolanda de Vries, Hein G Gooszen, Cornelis JHM van Laarhoven

**Affiliations:** 1 Department of Surgery of the Radboud University Nijmegen Medical Centre, Nijmegen, The Netherlands; 2 Department of Medical Psychology, Tilburg University, Tilburg, The Netherlands; 3 Department of Surgery of the University Medical Center, Utrecht, The Netherlands; 4 Department of Surgery, St. Elisabeth Hospital, Tilburg, The Netherlands

## Abstract

**Background:**

Cholecystectomy causes considerable financial burden on society with a major part caused by sick-leave. There are wide variations in duration of sick-leave. The aim of our study was to identify all aspects that influence the moment of return to work by using focus groups and to compare responses from patients and physicians.

**Methods:**

A qualitative research design was planned using focus group discussions. Four focus group discussions were organized: two patient groups and two physician groups. Employed patients who had recovered after cholecystectomy were included in the patient groups. The physicians groups consisted of general practitioners, surgeons, and company physicians. Three investigators independently searched transcriptions of the sessions for all items relating to return to work. The importance of items and categories were assessed by determining frequencies.

**Results:**

In the patients groups physical limitations (35.3%) and individual patient factors (17.5%) were important factors in the duration of sick-leave, while influence or advice comprised only 8.4% of the items. In the physicians groups influence or advice (21.8%) and information-related factors (21.4%) were thought to be important categories.

**Conclusions:**

Physicians perceive their advices as an important factor in patients' duration of sick-leave. In contrast, patients seldom mention this factor and experience physical complaints as the major reason influencing the moment of return to work.

## Background

For about 100 years, open cholecystectomy (OC) was considered a safe standard [1]. Reduction in the length of the incision, known as small-incision cholecystectomy (SIC), with a concomitant reduction in postoperative morbidity, has been reported as early as the mid 1970's [2,3]. However, before the SIC could find general acceptance, the laparoscopic cholecystectomy (LC) was introduced in the late 1980's [4]. This procedure gained rapid and immense popularity [5] and became the surgical treatment of choice even though its superiority was not in evidence [6].

Both minimal invasive techniques (SIC and LC) are preferred over the open technique because of a quicker convalescence (hospital stay and return to work) [7,8]. As no significant differences between LC and SIC in primary outcome measures were found [9], secondary outcome measures should further decide on preferences.

The financial burden of cholecystectomy on society is considerable with over 60% of the total costs of employed patients caused by indirect costs related to sick-leave of employees [10]. As time before return to work ranges from 1 to 10 weeks, both in successful LC and successful SIC [11], apparently other unknown factors are involved. The question arising is who or what influences the moment of return to work?

To answer this question, we have to know a wide range of factors that influence the absence from work. In the literature, patients' expectations [12], low job satisfaction, physical effort at work, pain, patient's expectation of slow recovery, expectation of no financial loss [13], a longer period of work incapacity before the intervention, older age, and longer hospital stay [14], are factors already identified in extending sick leave. The impact of cultural differences on the moment of return to work was shown as well [15]. These studies examined the influence of specific factors on the moment of return to work. However, as far as we know no attempt has been made to determine a wide range of factors that are involved.

Focus group discussions appeared to be a reliable method of gathering qualitative information on a subject [16,17]. A focus group is a type of group interview with the primary goal to generate ideas about a particular issue. The reliance in focus groups is on the interaction between the various participants [16]. The dynamic interplay of participants replaces their interaction with the interviewer, leads to a greater emphasis on the participants' point of view [18], generates additional ideas in the group, and is the additional value of focus group discussions compared to individual (patient) interviews.

The aim of our study was to retrieve a wide range of aspects that influence the moment of return to work after cholecystectomy and to compare responses from patients and physicians. This was done using focus groups. We hypothesize that a physicians' advice is important in the decision to return to work.

## Methods

### Participants

We organized four focus group discussions: two patient groups (seven patients each) and two physician groups (seven and eight physicians). Patients were randomly sampled from the patients included in our randomized clinical trial on outcome after laparoscopic and small-incision cholecystectomy [11]. Approval from the Medical Ethics Committee was obtained (ISRCTN67485658; http://isrctn.org). The indication for cholecystectomy in all patients was symptomatic cholecystolithiasis. The results and postoperative outcome of the patients in our trial were in line with results in literature [11]. A paid job was an inclusion criterion in patient groups. A second inclusion criterion was that patients should have had their cholecystectomy at least six months earlier to guarantee full recovery. In both patient groups, there were patients operated on by three techniques: laparoscopic, small-incision, and procedures converted to conventional cholecystectomy.

Physicians who come in contact with this type of patients and, thus, can influence in some way the moment of return to work of patients are: general practitioners, surgeons, and company physicians. Company physicians are doctors who independently advice patients and employers when work should be restarted or which alternative work may be performed by the patient when unable to restart their usual activities. All three types of physicians were present in both physician groups. Physicians were randomly sampled from a list representing the hospitals' affiliation area. They were invited to participate in the focus groups according to availability. These physicians were not physicians for these particular patients.

In total four focus groups were run, two patients groups and two physicians groups. One patients' focus group comprised of 4 men and 3 women and the other patients' focus group consisted of 1 man and 6 women. All patients had their cholecystectomy at least six months earlier. Participants in the first physicians' focus group were two company physicians, three general practitioners, and two surgeons. In the second physicians focus group there were two company physicians, four general practitioners, and two surgeons. Informed consent was obtained from all participants.

Compared with the second patients' focus group, in the first patients' focus group more patients had remaining complaints and symptoms. However, the items mentioned in these two focus groups were the same. Therefore, the three investigators felt that saturation was reached after only two sessions. The same occurred in the physicians focus groups.

### Method of group discussion

The focus groups were run by the authors and all investigators were present in all four focus group sessions. In accordance with focus group methodology, the role of the investigator leading the focus group was restricted to procedural issues (e.g. making sure that every participant had the chance for expression of his/her opinion) and posing the two opening questions in order to let the interaction between participants dominate the discussion.

The two opening questions in the patients' focus groups were: (1) how did the patient experience his/her cholecystectomy, and (2) who or what factors did influence the moment of return to work.

The two opening questions in the physicians' focus groups were: (1) how do the physicians think that patients experienced their cholecystectomy, and (2) who or what do physicians think influences the moment of return to work in patients.

The patient group discussion was started by the opening question how patients had experienced and felt about their operation. At the end of a discussion around a question, the investigator summarized the items that were mentioned and asked patients if there were items they could think of that had not yet been mentioned. Consequently, the other opening question was posed.

### Data recording

During the discussions, items were noted on a flip-over by one of the investigators. In addition, the sessions were audiotaped with permission from the patients. These tapes were subsequently verbatim transcribed.

### Assessing the number of focus groups

The number of focus group sessions was determined by saturation, i.e., when in another group no new items are mentioned by the participants in comparison to a previous similar focus group. When saturation is reached, the number of focus groups is considered to be adequate.

### Analysis

Analysis of results in qualitative research is completely different from classical statistical analysis of quantitative data. In our study, initially the transcriptions were searched for all possible items and factors and also items noted on the flip-over were added to the listed items from the transcriptions. Subsequently, by analyzing these items, main categories were determined and all items were classified into a main category independently by three investigators. Disagreements were solved in consensus. In this way, bias caused by analyzing and interpreting data was minimized by comparing these independently obtained results. Factors were clustered in subcategories after consensus. Then, the importance of the separate items was assessed by determining the frequency in which they were mentioned. The frequency of an item was used as a proxy for importance. After assessing the frequencies of each item, the importance of an individual item as well as the importance of a main group could be determined.

## Results

After transcription of the tapes and checking the flip-overs, eight main categories were defined: 'physical', 'hospital stay related', 'home', 'work-related', 'influence or advice (including expectations) of others', 'patients' expectations or individually determined factors', 'information', and 'other'. Consequently, items relating to the same subject were summed, leading to subcategories.

### Patients' focus groups

Results of the patients' focus groups are shown in table 1. All items of the two groups were combined and led to 309 items. A total of 23 items were irrelevant to return to work (e.g. a patient mentioning that it took a long time before the diagnosis was set), resulting in the 286 items that are listed (Table 1). Physical limitations (35.3%), individual patient factors (17.5%), hospital-related factors (16.4%), and work-related factors (16.1%) were important factors in time to return to the job. Influence or advice comprised only 8.4% of the items mentioned in the decision to resume the job. Home factors (4.2%) or information related factors (2.1%) were not experienced by patients to be important reasons for delaying or resuming work activities.

**Table 1:** Score of items relating to return to work in patients focus groups.

Physical factors	101 (35.3%)	Patients' expectations or individually determined factors	50 (17.5%)	Hospital related factors	47 (16.4%)
pain	15	consideration of being operated upon	7	good follow-up care	9
open wound	11	fear of the operation	6	earlier discharge	5
food related complaints	11	an individual's willingness to resume work	4	delayed discharge	5
general physical complaints	11	a person's character	3	operative technique	5
change for the better	9	individual differences	2	operation delay	4
wound pain	7	recovery is disappointing compared to others	3	postoperative information	3
diarrhea	6	differences in experiencing pain	2	physicians with limited time	3
insomnia	6	experiences in the past	1	Gallbladder condition	3
shoulder pain	5	other individual circumstances	3	visit to outpatients' clinic	3
lack of endurance	5	self determination	1	anesthesia	3
abdominal colic's	5	scar	4	operating surgeon dependent	2
gastrointestinal complaints	4	nervous disposition	2	waiting list	1
infection	3	relaxed attitude	3		
tiredness	3	disappointing	3		
		fear for resuming work activities	1		
		other	5		

**Work-related factors**	**46 (16.1%)**	**Influence, expectations or advice by thirds**	**24 (8.4%)**	**Home factors**	**12 (4.2%)**

type of work	11	discouraged by others	5	children	8
adapted work activities	8	pressure by employer	2	housekeeping	2
work with lifting activities	6	expectation of company physician	1	getting bored at home	1
part-time work	3	advice of company physician	1	gender differences	1
discouraged by employer	3	other factors related to company physician	2		
work atmosphere	2	pressure by company physician	1		
influence of temperature on wound	2	advice of surgeon	2		
possibility to return home early	2	advice of any other physician	1		
				
company physician related	1	financial pressure	1	**Information related factors**	**6 (2.1%)**
				
autonomy	1	structure of health care	1		
being in contact with the company	1	society's expectations	1		
continuity at work	1	people's expectations	2		
no use of a partly recovered colleague	1	advise of others	1		
				
independent (own store)	1	taking others into consideration	1	**Other**	**0**
				
relation with employer	1	expectations of children	2		
other	3				

Within the physical factor, pain (14.9%), an open wound (10.9%), food-related complaints (10.9%), wound complaints (10.9%), and general health related complaints (10.9%) were mentioned most frequently. A reduction in physical complaints after cholecystectomy was mentioned by 8.9% as a positive factor for return to work. Other factors were: wound pain (6.9%), diarrhea (5.9%), and insomnia (5.9%).

In the individual patient factor, patients mentioned that they had to be careful to resume their work as a simple consequence of having surgery (14%). Differences in the type of person (6%) and having fear for the operation (12%) were important reasons as well.

In hospital related factors, patients mentioned a positive follow-up care after the operation (19.1%), earlier (10.6%) or delayed (10.6%) discharge and the type of surgery (10.6%) to be important reasons.

In the work related factors, the type of work (23.9%), adapted work (17.4%) and work requiring weight lifting (13%) were most important.

"... initially I assisted in administrative activities, which is completely different from my usual activities on the job ..."

The main category 'influence or advice' was mentioned in 8.4% to be important and within this main category, the influence or advice of a physician was the reason in 33.3%. Sometimes patients wanted to work, but others advised them not to (20.8%).

"... the company physician advised not to resume work and to take it easy, just because I had abdominal surgery ..."

### Physicians focus groups

Results of the physicians' focus groups are shown in table 2. All items of these two groups were combined and led to 280 items. A total of 28 items were irrelevant to return to work (e.g. someone describing situations in other countries), resulting in the 252 items that are listed (Table 2). Influence or advice by others (21.8%) and information-related factors (21.4%) were the two most important categories in time to return to the job in the physicians' groups. Work-related factors (16.3%), individual patient factors (13.9%) and hospital-related factors (12.3%) were assessed less important in the decision to return to work. Physical factors (7.9%) and home factors (1.6%) were not experienced by physicians to be important reasons for patients in delaying or resuming their work activities.

**Table 2:** Score of items relating to return to work in physicians focus groups.

Influence or advice (including expectations) by thirds	55 (21.8%)	Information related factors	54 (21.4%)	Work-related factors	41 (16.3%)
society's expectations	10	lack of information	13	type of work	7
structure of health care	8	lack of guidelines	10	initiatives of the employer	6
advise of others	7*	a structure for communication	9	possibility to return home early	6
influences of cultural differences	7	pamphlet information	6	work atmosphere	4
people's expectations	6*	lack of advice	4	motivation	4
advice of general practitioner	6*	positive attitude	3	adapted work activities	4
advice of company physician	4*	differences in interpretation of information	2	financial aspects employer	3
advice of surgeon	3*	other	7	relation with employer	2
employer's expectations	2			commitment	2
other	2			independent (own store)	2
				other	1

**Patients' expectations or individually determined factors**	**35 (13.9%)**	**Hospital related factors**	**31 (12.3%)**	**Physical factors**	**20 (7.9%)**

individual differences	8	information about the operation	8*	pain	8
a person's character	7	type of operation	7	tiredness	6
motivation	5	visit to outpatients' clinic	7*	diarrhea	2
personal circumstances	5	information on recovery	4*	endurance	2
differences in experiencing pain	3	hospital stay	2	wound healing	2
an individual's interpretation of information	2	waiting list	2		
other	5	good follow-up care	1		

**Other**	**12 (4.8%)**	**Home factors**	**4 (1.6%)**		

legal aspects	4				
analgesics	4				
other	4				

* items somehow relating to information, not scored in the information category.

Within the 'influence or advice by others' category, physicians assessed that society expectations were the most important subcategory (18.2%). The structure of health care and financial arguments was thought to be important as well (14.5%). Other factors that were revealed: advices in general (12.7%), the role of the general practitioner (10.9%) and advices by societal contacts of patients (like neighbors) (10.9%). Cultural factors were thought to be important as well (12.7%).

In the (secondly most important) 'information-related items' category, physicians thought that lack of information (on expectations) to the patient is mainly responsible for delay in return to work (24.1%). Additionally, supplying information to patients by a pamphlet (11.1%) and a lack of guidelines (18.5%) were thought to be important as well.

"... do company physicians have guidelines on what to advice to patients considering the moment to return to work? No. And do general practitioners have guidelines? No. In the surgical world there are no guidelines or evidence on when to return to work either ... Actually, nobody knows what should be advised to patients ..."

Another important and time consuming issue in the discussions (and all physicians agreed on being important) was the lack of a structure to communicate between surgeons, general practitioners and company physicians (16.7%).

"... There is no contact and communication between surgeons, general practitioners and company physicians on what to advice to a patient on return to work. A structure for communication is necessary and currently missing, especially quick communication ..."

In the work-related category, the type of work (17.1%), initiative by the employer to contact the employee (14.6%), and flexibility (24.4%) (including adaptive activities (9.8%) and possibilities to return home at all times (14.6%)) were assessed important in the decision of patients to resume activities. Additionally the atmosphere at the job (9.8%) and an individual's motivation (9.8%) were assessed by physicians to play a role.

In the individual patient factors category, individual factors (22.9%), personality (20%), an individual's motivation (14.3%) and specific individual circumstances (14.3%) were the main subcategories.

In the hospital related category, physicians assessed that patients being informed by surgeons about operative findings (25.8%) and the process at the outpatients' clinic (22.6%) were the main subcategories. The type of operative technique (22.6%) was thought to be important as well.

Summarizing all items somehow relating to information or advice (including lack of information, lack of advice and incorrect advice), lead to a total of 99 items (39.3%).

## Discussion

The aim of our study was to retrieve a wide range of aspects that influence the moment of return to work after cholecystectomy and we hypothesized that a physicians' advice would be important in the decision when to return to the job. This qualitative research showed that physicians believe that their advices are most important. In contrast, patients mentioned this factor seldom and experience physical complaints as the major reason influencing their return to work. The hypothesis was rejected that a physicians' advice was important in a patients' decision to return to work. Our study showed that we need to pay more attention to the physical complaints after cholecystectomy. Surprisingly, guidelines concerning advice when to resume work activities and communication between physicians appear to be missing. Structured communication between physicians may supply the patient with an individual appropriate advice.

It has to be emphasized that we used a qualitative design and results should therefore be considered in an explorative perspective. Drawing quantitative conclusions from this study is inappropriate.

The inclusion criterion that patients had their cholecystectomy at least six months earlier may very well have resulted in recall bias. However, it was considered that a shorter period might have distorted results by information bias as a consequence of differences in the state of recovery in the participating patients. Patients might very well have overestimated the importance of current factors compared to factors that played a role in the past. Overall, we had the impression that patients remembered their recovery and sick-leave very well.

As far as we know, in literature only the influence of specific factors on the moment of return to the job was evaluated [12-15]. No attempt has been made thus far to identify a wide range of factors that influence the moment back to work. The factor 'an older age' was not identified, but all other factors mentioned in literature were included in our results. Work incapacity before the intervention was not the focus of this study.

We found several discordant findings between the patients and physicians focus groups. The first most striking difference between patients and physicians was that physicians think their advice is most important. Most of the items in the physicians group concerned advices to a patient to resume work. These physician advices were not mentioned in the patient group and, thus, time back to work is not related to this. There were some other items concerning possibilities of physicians believing that in some way the recovery of patients after surgery could be influenced (e.g. by preoperative expectations). Obviously, patients would not mention these items while they were not aware of the possibilities how their recovery could be influenced. This partly explained the difference between the two types of groups. It was also remarkable that some patients wanted to resume work, but they were advised by company physicians not to do so. Apparently, physicians were sometimes too careful.

Another major discordance was found in physical factors. Patients experienced physical complaints as the most important reason causing delay in return to work, while physicians did not assess the physical factors to be important. One could expect that pain in some way inhibits patients' return to work, but more surprising was the large number of gastro-intestinal complaints. Inability to eat, intestinal colic's and a disturbed defecation (i.e. diarrhea) were frequently mentioned reasons not to return to work. Being tired, inability to sleep and lack of general fitness were also frequently mentioned. A reduction in physical complaints after cholecystectomy was mentioned seldom by patients as a positive factor, while one would expect that disappearance of symptomatic cholecystolithiasis would be an important positive reason for resuming work activities.

Another problem that became obvious in the physicians groups concerned communication between various types of physicians. There appeared to be no communication between surgeons, general practitioners and company physicians relating to the moment of return to work of a patient. Company physicians sometimes wanted to ask a surgeon for advice, but writing, sending, answering and returning a letter takes up too long (several weeks) and was, therefore, impractical. Making a phone call often was a problem as well (e.g. because of performing surgery). In future, communication between surgeons and company physicians could combine technical information of the operation with the specific work-related knowledge of company physicians and might result in an appropriate advice for the individual patient.

One of the most surprising remarks in the physicians' groups came up when the existence of protocols was discussed. Each type of physician thought that the others had some kind of protocol about a standard period of sick leave and expected that their colleagues advised patients on when to return to work. However, no protocol or guidelines existed and no concrete advice was given to the patient. The main thing physicians told their patients in relation to time back to work was to listen to their body and to return to the job as soon as they felt well enough to do so. Advices varied and it seemed that every physician generates his or her own advice independent from each other. Company physicians and general practitioners gave advices without asking a surgeon for his opinion and a surgeon usually did not give any advice or simply referred to the company physician.

Since we included patients with a paid job in our focus group discussions it is not possible to conclude from our study on convalescence in patients without a paid job, although some factors might very well apply to these patient categories as well. We focused on individual aspects using a qualitative study design. The influence of other, non individual, more general factors like differences in social class and education level should be addressed by a quantitative study design.

This raises the question whether our results are generalisable to other general surgical procedures such as inguinal hernias, appendectomies and varicose surgery. We feel that many factors may very well be generalisable. Alternatively, other factors and especially incentives in the health care system may vary considerably from one place to another. The results may be different in countries without a Western European social security system where patients suffer financial loss during sick leave. Financial situation are different and the factors influencing return to work may therefore differ as well. However, future research is necessary to examine this.

## Conclusion

Physicians perceive of their advices as an important factor in patients' duration of sick leave. In contrast, patients seldom mention this factor and experience physical complaints as the major reason influencing the moment of return to work.

Attention has to be paid to patients' physical complaints after cholecystectomy as well as to the way information is supplied to the patient. More adequate information, generating guidelines on time back to work in general and improving communication between types of physicians, might result in an appropriate advice for every individual patient. Knowledge of factors influencing the moment of return to work may result in improvements of patients' recovery. Additionally, reductions in sick-leave and subsequently substantial savings in indirect costs may be reached.

## Competing interests

The authors declare that they have no competing interests.

## Authors' contributions

FK participated in the study concept and design, the acquisition of data, the analysis and interpretation of data, and the drafting of the manuscript. JdV participated in the study concept and design, the acquisition of data, the analysis and interpretation of data, the drafting of the manuscript, and critical revision of the manuscript. HGG participated in the study concept and design, the analysis and interpretation of data, and critical revision of the manuscript. CJHMvL participated in the study concept and design, the acquisition of data, the analysis and interpretation of data, and critical revision of the manuscript. All authors read and approved the final manuscript.

## Pre-publication history

The pre-publication history for this paper can be accessed here:

http://www.biomedcentral.com/1471-230X/10/12/prepub
